# Micronutrient intake inadequacies in different types of milk consumers in Indonesian children 1–5 years: dietary modeling with young child milk improved nutrient intakes

**DOI:** 10.3389/fnut.2023.1169904

**Published:** 2023-06-26

**Authors:** Diana Sunardi, Yulianti Wibowo, Tsz Ning Mak, Dantong Wang

**Affiliations:** ^1^Department of Nutrition, Faculty of Medicine, Universitas Indonesia – Cipto Mangunkusumo Hospital, Jakarta, Indonesia; ^2^Medical Nutrition Services, Nestle Indonesia, Jakarta, Indonesia; ^3^Nestle Institute of Health Sciences, Singapore Hub, Nestle Research, Singapore, Singapore; ^4^Nestle Institute of Health Sciences, Nestle Research, Lausanne, Switzerland

**Keywords:** nutrient intake, children under-five, young child milk, dietary modeling, inadequacy

## Abstract

**Background:**

Indonesian children under-five have a high prevalence of micronutrient deficiencies. Improving young child feeding practices may be the solution. Increasing the consumption of appropriate milk products could help to reduce nutrient inadequacy.

**Methods:**

The objective of this study was to assess nutrient inadequacy in Indonesian children to evaluate the potential improvement using dietary modeling analysis. Data from children aged 1–5 years from the Indonesian Individual Dietary Consumption Survey in 2014 were used in this analysis (*n* = 11,020). Diet modeling was conducted in two scenarios, substitution volume to volume and calories to calories.

**Results:**

The proportion of children consuming young child milk (YCM) was the highest compared to other milk types across all age groups, followed by condensed milk and cow’s milk. YCM, also called “Growing-Up Milk” (GUM), are marketed as a product specifically formulated for the nutritional needs of young children. YCM consumers had lower prevalence of inadequate intakes in iron, zinc, vitamins A, C and D across age groups when compared to condensed milk consumers. The prevalence of inadequate intakes of nutrients in condensed milk consumers was Vitamin A (67, 64%), folate (92, 91%), Vitamin D (87, 84%), iron (84, 76%), and zinc (76, 76%) in 1–2y and 3–4y, respectively. The substitution of condensed milk with a YCM reduced the prevalence of inadequate intakes of micronutrients, such as Vitamin A, vitamin D, folate, iron and zinc, which are important for immune function. YCM reduced the prevalence of inadequate intakes of micronutrients by 20–40% (Vit A and folate) and 40–50% (Vit D and zinc). The reduction of prevalence of inadequate iron intake was 31% in 1–2y and 63% in 3–4y.

**Conclusion:**

The prevalence of inadequate micronutrient intakes was high among children aged 1–5 years old in Indonesia. YCM consumers had better nutrient intake. The substitution of condensed milk with a YCM reduced the prevalence of inadequate of micronutrient intake. Thus, nutrient intakes could be improved by YCM consumption in 1–5 years old children in Indonesia, along with nutrition education on feeding practices.

## 1. Background

Indonesia has achieved several milestones in its developmental journey to a middle-income country. Decreases in children’s deaths and significant increases in primary school enrolment are among them. However, improvement in children’s nutritional status, has not been observed. Millions of Indonesian children and adolescents remain threatened by staggering rates of stunting and wasting and the ‘double burden’ of malnutrition where undernutrition and overnutrition co-exist. While poverty contributes to malnutrition, inadequate knowledge, and practices of childcaring and child feeding also sustain the high rates of malnutrition (1). Indonesia has made some progress toward achieving the target for stunting and wasting: however, 30.8 and 10.2% of children under 5 years of age were affected (2). Better dietary intake is one of the key aspects to improve the situation. A systematic review showed that in children under-5 years in Indonesia, micronutrient intake varied, for example iron ranged from 2.40 to 12.8 mg/d, zinc ranged from 1.20 to 5.53 mg/d, calcium ranged from 83 to 707.9 mg/d, vitamin A ranged from 53 to 1,335 ug/d, and vitamin D ranged 2.75–14.10 ug/d (3). Prevalence of micronutrient deficiency in children under-five was also reported in a national survey, such as anemia (urban 17.6% vs. rural 18.5%), iron deficiency (urban 4.6% vs. rural 8.8%), and vitamin D deficiency (urban 43% vs. rural 44.2%) (4). Although several good practices have been put in place, some critical ones are not fully implemented. These are related to dietary diversity, healthy feeding and hygiene practices. Poor feeding practices result in suboptimal dietary intake. The available data suggest that children’s consumption of nutrient rich foods was limited (5) and feeding practices are not optimal among children aged 6 months and above (6). Improving feeding practices is therefore crucial to tackle malnutrition among Indonesian young children.

Milk and dairy products are nutrient-dense foods, supplying energy and high-quality protein with a range of essential micronutrients (especially calcium, magnesium, potassium, zinc, and phosphorus) in an easily absorbed form for normal growth and development in children (7). Increasing the consumption of milk products among children may help to reduce nutrient inadequacy. To test this hypothesis, dietary modeling is a useful tool for assessing the theoretical nutritional impact of increasing dairy consumption in the study population. Dietary modeling is a statistical technique that has been increasingly applied to inform the development of dietary guidelines worldwide. In 2020, Dietary Guidelines for Americans Advisory Committee defined it as a method to understand how changes to the amounts or types of foods and beverages in a diet might impact meeting nutrient needs (8). Using dietary modeling, a study in Filipino children 1–5 years old suggested an improvement of macro- and micronutrient intakes by adding a serving of a milk product to the diets of children who did not meet their dairy intake recommendations (9). In the present study, we analyzed the nutrient intakes in Indonesian children aged 1–5 years who consumed different types of milk and applied dietary modeling approach to investigate the potential impact on nutrient intakes. This is, to our knowledge, the first such report for Indonesia.

## 2. Methods

The objective of this study was to assess nutrient inadequacy in children 1–5 years old in Indonesia and to evaluate the potential improvement in nutrient adequacy by dietary modeling analysis. Data of children aged 1–5 years from the Indonesian Individual Food Consumption Survey (IFCS) in 2014 (*n* = 11,020) were used for this analysis. This Survey was part of the Indonesian Total Dietary Intake Study (SDT 2014), providing quantitative information on food intake by households and individuals (10). Inclusion criteria is all children aged 12–60 months old, with the exclusion criteria, if the demographic and dietary intake were not complete.

The energy and nutrient intakes of each child were estimated based on a 1 day 24-h dietary recall using Nutri-Survey software (EBISpro, Germany). Milk consumers were defined based on the consumption of different types of milk, including young child milk (YCM), condensed milk or cow’s milk, by children that were recorded in the 24 h food recall in the SDT survey. The energy and nutrient intakes of children were compared among the different milk consumer groups defined above. The SDT 2014 classification approach of energy and protein intake status, and WHO estimated average requirements (EAR) for micronutrients were applied to define inadequate intakes in this study. A substitution approach was applied in diet modeling, that is, by substituting condensed milk with equal volume and isocaloric YCM. YCM also called “Growing-Up Milk” (GUM), are marketed as products specifically formulated for the nutritional needs of young children (11). Dancow 1+ and 3+ composition was used in the diet modeling as YCM examples for children aged 12–35 months and 36–60 months, respectively. Statistical analysis was done using SPSS software version 20. To investigate the potential difference in nutrient intake across age, the analyses were stratified by age group 12–23, 24–35, 36–47, and 48–60 months for nutrient adequacy analysis, and 12–35 and 36–60 months for dietary modeling, respectively. Differences in subject’s characteristics (gender, living area, SES, and mother’s education) were also tested. The Chi-square test was performed to test the statistical significance of characteristics by type of milk consumption group, Fisher’s exact test was used to compare the percentages of inadequate intake in YCM consumers to those of condensed milk and cow’s milk consumers, as well as to compare the prevalence of baselines to that of the modeling scenarios. The Wilcoxon signed-rank test was applied to compare the nutrient intake distribution (12).

## 3. Results

### 3.1. Subjects

This study assessed the dietary intakes of 11,020 children aged 1–5 years from the SDT survey. We found a higher proportion of children living in rural areas (57.1%) than in urban areas (42.9%), while the distribution of children by gender and age was similar. The detailed distributions of the subject’s characteristics distribution are shown in Table 1.

**Table 1:** Subject characteristics (*n* = 11,020).

Variable	Frequencies	%
Region		
Urban	4,731	42.9
Rural	6,289	57.1
Gender		
Boys	5,694	51.7
Girl	5,326	48.3
Age		
Months		
12–23	2,529	22.9
24–35	2,701	24.5
36–47	2,847	25.8
48–59	2,943	26.7

### 3.2. Subjects and households’ characteristics of consumers of different milk beverages

Based on the dietary recall, YCM, condensed milk and cow’s milk were the top three of dairy foods consumed by the study population. Hence, in this study, subjects were classified into four groups namely non-milk consumers (*n* = 6,283), YCM consumers (*n* = 3,138), condensed milk consumers (*n* = 991), and cow’s milk (pasteurized milk) consumers (*n* = 608; Table 2). Approximately half of the children (46–57%) did not consume any milk product between 12 and 35 months and 48–60 months. However, only 18% of the children did not consume milk products between 36 and 47 months. Among milk consumers, the proportion of children who consumed YCM was the highest across age groups, followed by condensed milk and cow’s milk. Children aged 24–35 and 36–47 months had, respectively, the lowest (33.4%) and highest percentage of YCM consumption (57.7%), respectively. The proportion of children consuming condensed milk showed an increasing trend from 12–23 to 36–47 months, and slightly decreased between 48 and 60 months. The proportion of children consuming cow’s milk was doubled in children aged 24–35 months compared to 12–23 months and remained stable at around 8%. A similar trend was observed for condensed milk intake (Table 2).

**Table 2:** Subjects and household characteristic by type of milk consumption.

Characteristic		Non-milk consumer	YCM	Condensed milk	Cow’s milk
		*n* = 6,283	*n* = 3,138	*n* = 991	*n* = 608
Gender	Boys	51.7%	50.7%	53.4%	52.6%
	Girl	48.3%	49.3%	46.6%	47.4%
	*p-*value	*P*b > 0.05	*P*a > 0.05	*P*a > 0.05, *P*b > 0.05	*P*a > 0.05, *P*b > 0.05
Age	12–23 m	54.80%	35.3%	6.5%	3.4%
	24–35 m	45.80%	33.4%	13.5%	7.3%
	36–47 m	18.50%	57.7%	15.5%	8.3%
	47–59 m	56.80%	21.9%	13.3%	8.0%
	*P-*value	*P*b < 0.01	*P*a < 0.01	*P*a < 0.01, *P*b < 0.01	*P*a < 0.01, *P*b < 0.01
Location	Urban	33%	60%	44%	54%
	Rural	67%	40%	56%	46%
	*P-*value	*P*b < 0.01	*P*a < 0.01	*P*a < 0.01, *P*b < 0.01	*P*a < 0.01, *P*b < 0.01
SES	Low	69.6%	40.0%	60.9%	48.4%
	Middle	27.2%	46.7%	34.8%	43.2%
	High	3.3%	14.4%	4.3%	8.4%
	*P-*value	*P*b < 0.01	*P*a < 0.01	*P*a < 0.01, *P*b < 0.01	*P*a < 0.01, *P*b < 0.01
Mother education	No education	6.2%	3.1%	4.1%	3.1%
	Not graduated elementary	12.4%	8.2%	9.2%	8.2%
	Elementary	34.0%	25.5%	34.7%	33.7%
	Junior high	20.6%	20.4%	23.5%	21.4%
	Senior high	20.6%	30.6%	21.4%	25.5%
	Diploma	3.1%	5.1%	4.1%	4.1%
	University	3.1%	7.1%	4.1%	4.1%
	*P*-value	Pb < 0.01	Pa < 0.01	Pa < 0.01, Pb < 0.01	Pa < 0.01, Pb < 0.01

Chi-square test was performed to test the statistical significance among the groups. Pa, non-milk consumer group was used as the reference group; Pb, YCM group was used as the reference group.

Overall, 67% of the non-milk consumers were lived in rural areas. Compared to rural areas, a higher proportion of children from urban areas consumed YCM (60%) and cow’s milk (54%,), whereas condensed milk was mostly consumed by children in rural areas (56%). The type of milk consumption differed by socioeconomic status (SES). In this study, we found that a higher proportion of non-milk consumers were from lower SES families, and that a higher proportion of children from higher SES families consumed YCM. Mothers of children in the non-milk consumer group tended to have lower education levels than those of the milk consumer groups. Conversely, mothers of children in the YCM consumer group tended to have higher educational levels than those in other milk consumer groups (Table 2).

### 3.3. Nutrient intake according to types of milk consumption

**Figure 1:**
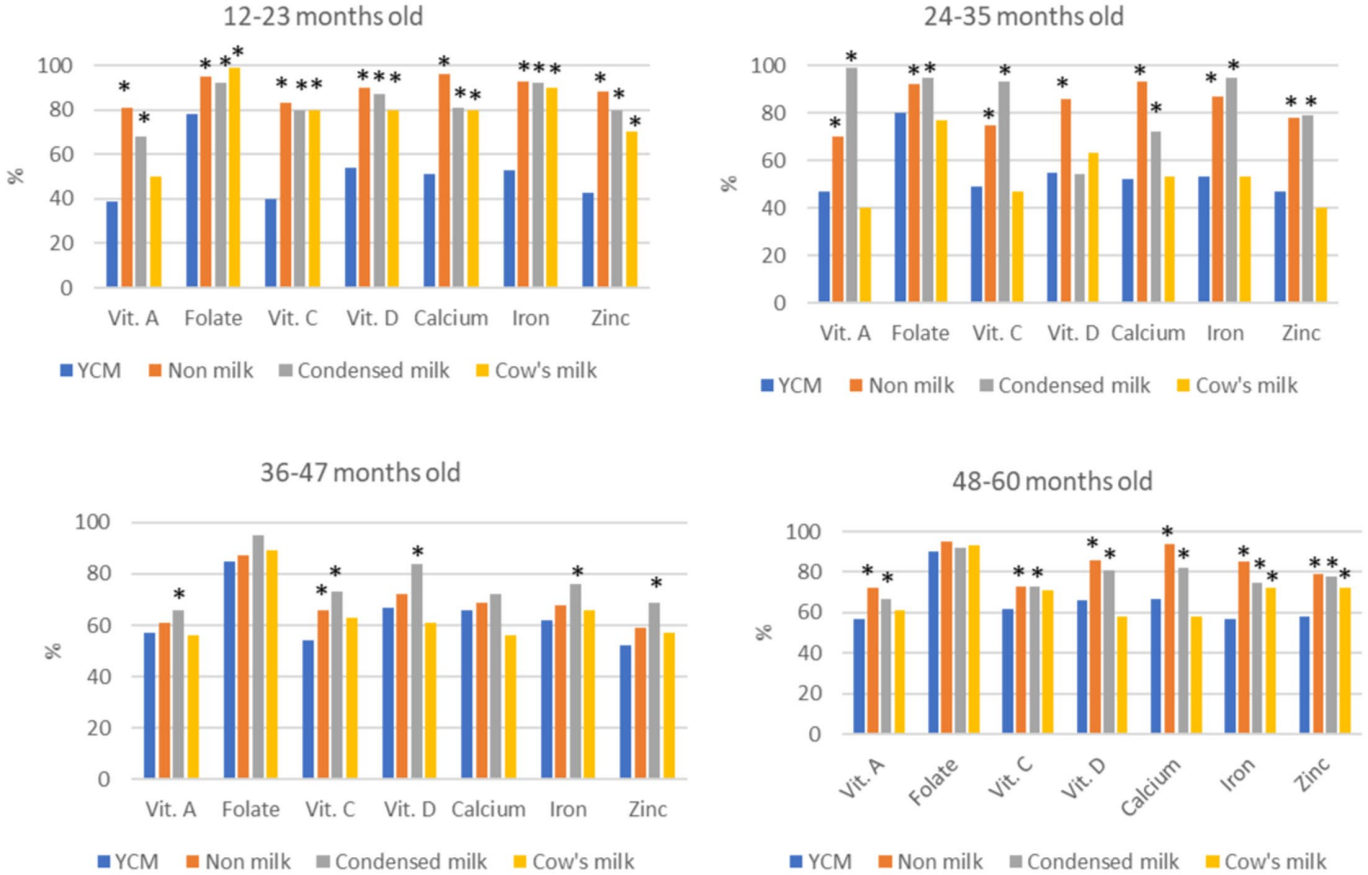
Percent of children with inadequate micronutrient intakes by types of dairy products in each age group. YCM group was used as the reference group, Fisher exact test was performed to compare the percentages of inadequate intakes in YCM consumers to that of condensed milk and cow’s milk consumers. *p < 0.05 after applying Bonferroni correction for multiple comparison.

More than 80% of the children were below the recommended energy intake in this population, and no difference was found among children who consumed different types of milk (not shown). YCM consumers had a lower prevalence of inadequate intake of iron, zinc, vitamins A, C, and D across age groups when compared to non-milk consumers, and condensed milk consumers. When compared to cow’s milk consumers, the prevalence of inadequate intake of iron, zinc, folate, vitamins C and D was lower between 12 and 23 months, but no significant difference was found in the older groups (Figure 1).

### 3.4. Diet modeling

Condensed milk was the second-most consumed milk in the study population. To investigate the potential impact of YCM consumption compared to condensed milk consumption, a diet modeling analysis was performed using a substitution approach. Two scenarios were evaluated among condensed milk consumers. First, substitution of condensed milk with the YCM, on an equal volume basis. Second, substitution of condensed milk with isocaloric YCM. For children aged 12–35 months and 36–60 months, Dancow 1+ and Dancow 3+ compositions were used in the analysis, respectively, as described in the Method. Tables 3, 4 showed that in both scenarios, the substitution of condensed milk with YCM reduced the prevalence of inadequate intakes of micronutrients, such as Vit A, iron, folate and zinc that are important for the maintenance of normal immune function.

**Table 3:** Modeling for nutrient intakes and inadequacy among condensed milk consumers (1–2y, *N* = 507).

		Mean	SD	P25	P50	P75	p1	Reference values (12–35 m)	% Children below reference value	p2
Vit. A	Baseline	319.5	671.2	17.6	123.2	401.2		286	67%	
	Scenario 1	458.1	687.6	161.7	277.1	528.1	<0.01	286	52%	<0.01
	Scenario 2	467.9	710.8	168.5	283.4	532.8	<0.01	286	50%	<0.01
Folate	Baseline	46.5	66.4	9.6	26.1	54.7		120	92%	
	Scenario 1	104.5	95.6	48.3	76.4	125.9	<0.01	120	73%	<0.01
	Scenario 2	106	98.2	49	76.8	127.8	<0.01	120	73%	<0.01
Vit. D	Baseline	2.1	4.9	0	0.3	2.2		5	87%	
	Scenario 1	8.4	9.6	3.4	5.7	10.6	<0.01	5	47%	<0.01
	Scenario 2	8.7	9.9	3.6	5.8	11	<0.01	5	47%	<0.01
Calcium	Baseline	335.3	417.3	146.4	242.7	402		417	77%	
	Scenario 1	530.9	497	267.3	420.2	647.2	<0.01	417	49%	<0.01
	Scenario 2	544.9	509.3	275.3	433.9	653.1	<0.01	417	49%	<0.01
Iron	Baseline	17.8	107.9	1.7	2.8	4.5		5.8	84%	
	Scenario 1	20.6	107.8	3.6	5.2	8	<0.01	5.8	58%	<0.01
	Scenario 2	20.7	107.8	3.6	5.3	8.1	<0.01	5.8	58%	<0.01
Zinc	Baseline	2.6	2.3	1.3	2.2	3.3		3.4	76%	
	Scenario 1	4.4	3.2	2.6	3.7	5.3	<0.01	3.4	44%	<0.01
	Scenario 2	4.5	3.3	2.6	3.7	5.4	<0.01	3.4	44%	<0.01

The intakes of different nutrients were compared to the baseline levels after substitution. Scenario 1, Substituted condensed milk with the same volume of YCM (volume to volume); Scenario 2, Substituted condensed milk with isocaloric YCM (kcal to kcal); SD, standard deviation; p1, *p*-value of Wilcoxon signed-rank test; p2, *p*-value of Fisher’s exact test.

**Table 4:** Modeling for Nutrient intakes and inadequacy among condensed milk consumers (3–4y, *N* = 797).

		Mean	SD	P25	P50	P75	p1	Reference values (36–47 m)	Reference values (48–60 m)	% Children below reference value	p2
Vit. A	Baseline	370.2	733.1	23.5	167.5	448.4		286	321	63.70%	
	Scenario 1	638.7	1846.2	199.8	375.3	614.9	<0.01	286	321	39.60%	<0.01
	Scenario 2	569.2	868	196.2	361.8	605.8	<0.01	286	321	40.30%	<0.01
Folate	Baseline	59.3	82.2	14.8	33.8	68.2		120	160	90.70%	
	Scenario 1	155.1	458	64.4	101.5	155	<0.01	120	160	68.10%	<0.01
	Scenario 2	136.9	159.2	63.2	99.5	152.3	<0.01	120	160	69.00%	<0.01
Vit. D	Baseline	2.4	4.3	0	0.5	3.2		5	5	84.10%	
	Scenario 1	13.1	52.5	4.4	7.8	12.2	<0.01	5	5	26.90%	<0.01
	Scenario 2	10.9	16.5	4.2	7.7	12.1	<0.01	5	5	32.00%	<0.01
Calcium	Baseline	389.9	481.8	182.8	278.8	433.2		417	500	76.80%	
	Scenario 1	711.9	2170.1	328.5	489	720.2	<0.01	417	500	43.40%	<0.01
	Scenario 2	624.2	711.2	322.7	481.2	704.6	<0.01	417	500	45.40%	<0.01
Iron	Baseline	24.2	160.5	2.4	3.7	5.9		5.8	6.3	75.80%	
	Scenario 1	30.9	163.2	5.8	8.4	12	<0.01	5.8	6.3	27.40%	<0.01
	Scenario 2	29.6	160.5	5.7	8.2	11.9	<0.01	5.8	6.3	28.60%	<0.01
Zinc	Baseline	3.1	3.9	1.6	2.5	3.9		4	4	76.20%	
	Scenario 1	6.8	19.1	3.5	5	7	<0.01	4	4	32.70%	<0.01
	Scenario 2	6.1	6.8	3.4	4.9	6.8	<0.01	4	4	34.90%	<0.01

The intakes of different nutrients were compared to the baseline levels after substitution. Scenario 1, Substituted condensed milk with the same volume of YCM (volume to volume); Scenario 2, Substituted condensed milk with isocaloric YCM (kcal to kcal); SD, standard deviation; p1, *p*-value of Wilcoxon signed-rank test; p2, *p-*value of Fisher’s exact test.

## 4. Discussion

There are studies on children’s nutrient intakes in Indonesia, but an adequate summary of nutrient inadequacies was challenging for Arini and colleagues to provide, due to the heterogenicity in subject characteristics among studies (3). In this study, data were derived from Indonesian 2014 Food Intake Survey, a national representative survey. This is an appropriate dataset to assess the nutrient intakes in Indonesia with 11,020 children aged 1–5 years, with representative distribution in location, gender, and age groups (10).

Meeting nutritional requirements is often a challenge, especially in the period in which children’s diets become diversified due to the introduction of solid foods (13). In general, milk and milk products should be important parts of children’s diet as they are rich in macronutrients and micronutrient such as protein, calcium, magnesium, phosphorus, zinc, potassium, vitamin D, vitamin A, riboflavin, and vitamin B-12. The SEANUTS study (The Southeast Asian Nutrition Survey), conducted in children 1–12 years of age, found that dairy consumption was positively associated with nutritional status and consumers were less likely to be deficient in vitamin A or insufficient in vitamin D (14).

However, there are various types of milk available in the market and their nutritional value varies considerably (15). Condensed milk is consumed by people at different ages and can be found easily in many parts of the world, including Indonesia. Condensed milk is a dairy product in the form of a viscous liquid obtained by removing some part of the water from a the mixture of milk and sugar or by other processes so that it reaches a certain level of concentration. Sugar was then added to prevent damage to the product. This product is usually used as a topping for various desserts or is mixed with beverages. Because of the high content of sugar used as a preservative (estimated close to 50% of the total calories), normally condensed milk should not be offered to children <12 months and older as a substitute for breast milk, YCM or cow’s milk. However, improper utilization of condensed milk has been reported previously in Indonesia (15–19). A study in Yogyakarta (a province in Indonesia) showed that, although milk consumption among preschool children was dominated by YCM (48.6%), there was still a significant proportion of children consuming condensed milk (29.3%) (14). Our results were similar to those of the above study, in that the highest proportion of milk consumers in 1–5 years old children consumed YCM, followed by condensed milk and cow’s milk.

The ESPGHAN Committee on Nutrition suggested that YCM can be considered as an option to increase nutrient intakes (20). A study in 300 healthy children aged 1–3 years in Jakarta observed that a daily consumption of two services YCM (≥300 mL/day) could be considered as a potential preventive measure for stunting in children (adjusted OR = 0.28, 95%CI 0.13–0.63) (21). The YCM was reported as a beneficial contributor of bioavailable calcium and potassium, and had a specific stimulating effect on linear growth and IGF-1 (insulin-like growth factor 1) levels in the children (22, 23). The study in Yogyakarta also reported the benefit of YCM consumption in a population where the prevalence of iron (low ferritin), zinc, and iodine deficiencies, vitamin D insufficiency and deficiency were still high, and the lowest prevalence of nutrient deficiencies was found in YCM consuming group (15). The result of the present analysis support the Yogyakarta study. We also found that YCM consumers had lower prevalence of inadequate intakes of iron, zinc, vitamins A, C and D across age groups when compared to condensed milk consumers. Substitution of condensed milk with YCM in our modeling analysis resulted in a significant increase of nutrient intake, showing that low nutrient intake could be significantly improved by changing the low-nutrient dense milk (condensed milk) with YCM. The results of this modeling were aligned with those of studies in the UK, China and the Philippines (12, 23, 24). The simulation scenarios study in the UK suggested more specifically that the consumption of YCM, instead of whole cow’s milk, might lead to improvements in nutrient intake, particularly for protein, fat quality, vitamin D and iron (24). The study conducted in China showed that YCM contributed significantly to the nutrient intakes of its consumers and the YCM consumers had higher nutrient intakes than non-consumers in many nutrients, such as iron, calcium, vitamin C, vitamin D, vitamin E, vitamin B6, and folate, all of which are important for growth and immune function (12). The dietary modeling in children aged 1–5 years in the Philippines, showed an improvement in minerals and vitamin intakes especially when a serving of YCM was added to child total diet (25). Finally, a randomized controlled trial study in New Zealand showed that, compare to unfortified cow’s milk consumers, YCM consuming group had lower prevalence of iron and vitamin D deficiencies in children aged 1–2 years (26). Results of these studies all point to the same direction, i.e., diet quality can be improved by YCM consumption. These studies indicate the importance of choosing the right milk, suggesting potential measures to ameliorate micronutrient deficiency through consumption the proper type of milk and establishing heathy dietary habits.

The present study is the first to analyze the impact of different milk consumptions on nutrient intake using data from a national representative survey in Indonesia. The dietary modeling approach was also applied for the first time and the results highlighted the advantage of YCM consumption in the study population. The study had some limitations. First, this was a cross sectional study, and no causal relationship could be inferred. Second, we found differences among different milk consumer groups in dwelling location, SES, and mother education levels. A multivariate analysis is required to further reveal the influence of different milk consumption. Third, the modeling approach described a potential theoretical impact; other aspects, such as cost and eating habits, need to be considered in future studies.

## 5. Conclusion

In this study, we found that in children 1–5y in Indonesia, the most consumed milk type was YCM, followed by condensed milk and cow’s milk, which could be related to SES. The prevalence of inadequate micronutrient intake was still high in the study population. Compared to condensed milk and cow’s milk consumers, YCM consumers had improved micronutrient intake, including vit A, iron, and zinc. Substitution of condensed milk with YCM in dietary modeling showed a reduction in the prevalence of inadequate intakes of micronutrients, such as Vit A, Vit D, iron, folate, and zinc, which are important for the maintenance of normal immune function. Thus, nutrient intake could be improved by YCM consumption in 1–5 years old children in Indonesia, along with nutrition education on food diversity.

## Data availability statement

The original contributions presented in the study are included in the article/Supplementary material, further inquiries can be directed to the corresponding author.

## Ethics statement

Ethical approval number LB.02.01/5.2/KE.189/2014 were received from the Ethical Commission Health Research, Health Research and Development Commission in Indonesian Ministry of Health. Written informed consent to participate in this study was provided by the participants’ legal guardian/next of kin.

## Author contributions

DS, YW, TM, and DW participated in the proposal development and the manuscript writing. DS conducted the data analysis. All authors contributed to the article and approved the submitted version.

## Funding

This study was funded by the Nestle Research. Nestle Research Center Team was involved in the study design and the development of statistical analysis plan, however, it was not involved in data analysis or result reporting.

## Conflict of interest

DS is a lecturer at the Faculty of Medicine, Universitas Indonesia, and a Physician Clinical Nutrition Specialist at Cipto Mangunkusumo Hospital. YW, TM, and DW are part of Nestle Scientific team.

## Publisher’s note

All claims expressed in this article are solely those of the authors and do not necessarily represent those of their affiliated organizations, or those of the publisher, the editors and the reviewers. Any product that may be evaluated in this article, or claim that may be made by its manufacturer, is not guaranteed or endorsed by the publisher.
