# Corrigendum: Micronutrient intake inadequacies in different types of milk consumers in Indonesian children 1–5 years: dietary modeling with young child milk improved nutrient intakes

**DOI:** 10.3389/fnut.2023.1258584

**Published:** 2023-07-26

**Authors:** Diana Sunardi, Yulianti Wibowo, Tsz Ning Mak, Dantong Wang

**Affiliations:** ^1^Department of Nutrition, Faculty of Medicine, Universitas Indonesia – Cipto Mangunkusumo Hospital, Jakarta, Indonesia; ^2^Medical Nutrition Services, Nestle Indonesia, Jakarta, Indonesia; ^3^Nestle Institute of Health Sciences, Singapore Hub, Nestle Research, Singapore, Singapore; ^4^Nestle Institute of Health Sciences, Nestle Research, Lausanne, Switzerland

**Keywords:** nutrient intake, children under-five, young child milk, dietary modeling, inadequacy

In the published article, there was an error in the article title. Instead of “Micronutrients intake inadequacy in different types of milk consumers in Indonesian children 1–5 years: dietary modeling with young child milk improved nutrients intake”, it should be “Micronutrient intake inadequacies in different types of milk consumers in Indonesian children 1–5 years: dietary modeling with young child milk improved nutrient intakes”.

The authors apologize for this error and state that this does not change the scientific conclusions of the article in any way. The original article has been updated.

