# Supplementary material for: Micronutrient intake inadequacies in different types of milk consumers in Indonesian children 1–5 years: dietary modeling with young child milk improved nutrient intakes
Source: Front Nutr. 2023 Jun 26;10:1169904. doi: 10.3389/fnut.2023.1169904 (PMC10411456; doi:10.3389/fnut.2023.1169904)
Supplement: Supplementary file 1 [file Table_1.DOCX]

Appendix

Table of Young Child Milk* Composition for Modelling

| Parameter | | 1+ | | 3+ | |
| --- | --- | --- | --- | --- | --- |
|  |  | **UoM** | **per 100g** | **UoM** | **per 100g** |
|  |  |  |  |  |  |
| ENERGY (KCAL) 449 | | kcal | **70.10656** | kcal | **74.13604** |
| Energy | | kJ | **293.3259** | kJ | **310.1852** |
| Protein | | g | **2.103197** | g | **2.35533** |
| Fat | | g | **2.804262** | g | **2.858883** |
|  | |  |  |  |  |
| Ca (Calcium) | | mg | **96.59126** | mg | **113.7056** |
| Fe (Iron) |  | mg | **0.841279** | mg | **1.674721** |
| Zn (Zinc) |  | mg | **0.592011** | mg | **0.990863** |
| Vit A |  | µgRE | **71.19711** | µgRE | **90.96447** |
| Vitamin D |  | µgD | **2.025301** | µgD | **2.761421** |
| Folic Acid |  | µg | **16.87231** | µg | **23.5533** |

*Dancow 1+ (YCM for 1 y and above) and 3+(YCM for 3 y and above)

Composition is based on current local version
